# Indole-3-carbinol protects against cisplatin-induced acute nephrotoxicity: role of calcitonin gene-related peptide and insulin-like growth factor-1

**DOI:** 10.1038/srep29857

**Published:** 2016-07-15

**Authors:** Reem N. El-Naga, Yasmen F. Mahran

**Affiliations:** 1 Department of Pharmacology and Toxicology, Faculty of Pharmacy, Ain Shams University, Cairo, Egypt

## Abstract

Nephrotoxicity associated with the clinical use of the anticancer drug cisplatin is a limiting problem. Thus, searching for new protective measures is required. Indole-3-carbinol is a powerful anti-oxidant, anti-inflammatory and anti-tumor agent. The present study aimed to investigate the potential protective effect of indole-3-carbinol against cisplatin-induced acute nephrotoxicity in rats. Rats were pre-treated with 20 mg/kg indole-3-carbinol orally before giving cisplatin (7 mg/kg). Cisplatin-induced acute nephrotoxicity was demonstrated where relative kidney weight, BUN and serum creatinine were significantly increased. Increased oxidative stress was evident in cisplatin group where GSH and SOD tissue levels were significantly depleted. Also, lipid peroxidation and NOX-1 were increased as compared to the control. Additionally, renal expression of pro-inflammatory mediators was induced by cisplatin. Cisplatin-induced cell death was shown by increased caspase-3 and decreased expression of EGF, IGF-1 and IGF-1 receptor. Nephrotoxicity, oxidative stress, inflammation and apoptotic effects induced by cisplatin were significantly ameliorated by indole-3-carbinol pre-treatment. Besides, the role of CGRP in cisplatin-induced nephrotoxicity was explored. Furthermore, cisplatin cytotoxic activity was significantly enhanced by indole-3-carbinol pre-treatment *in vitro*. In conclusion, indole-3-carbinol provides protection against cisplatin-induced nephrotoxicity. Also, reduced expression of CGRP may play a role in the pathogenesis of cisplatin-induced renal injury.

Cisplatin (*cis-* diaminedichloroplatinum, CDDP) is an effective chemotherapeutic agent used to treat various types of cancer^1^. Indeed, nephrotoxicity have been shown to be the main dose-limiting side effect that occurs in nearly 30% of the patients^2^,^3^ Cisplatin-induced renal insufficiency is demonstrated by increased serum creatinine and blood urea nitrogen levels, decreased renal blood flow, hypomagnesemia, hypocalcemia and proteinuria that reflects tubular dysfunction^4^,^5^. Moreover, repeated cumulative cisplatin dosing may result in chronic renal failure^3^,^5^,^6^. Therefore, searching for effective preventive strategies is an important approach.

The pathogenesis of cisplatin-induced nephrotoxicity was found to be multifactorial^3^,^7^. It was shown that cisplatin is transported by special membrane transporters into renal cells where it undergoes activation into more toxic products^8^,^9^. Additionally, cisplatin-induced renal injury is partially mediated via forming adducts with nucleophilic macromolecules such as DNA, RNA and proteins^10^. There is strong evidence that activation of a multiple cell death and intervention with survival pathways are involved^11^. Indeed, oxidative stress and inflammation play a major role in cisplatin nephrotoxicity. Over the last decade, many pro-inflammatory mediators were shown to be involved in renal injury such as interleukin-1beta (IL-1β), tumor necrosis factor-alpha (TNF-α) and nuclear factor-kappa B (NF-kB)^12^,^13^,^14^. Accordingly, using anti-oxidant/anti-inflammatory agents may present promising chemopreventive strategy against cisplatin-induced nephrotoxicity.

Indole-3-carbinol is a natural glucosinolate breakdown product found in cruciferous vegetables^15^. It was shown that indole-3-carbinol has the ability to increase the activity of some anti-oxidant enzymes such as hemoxygenase-1 and glutathione transferase^16^. Besides, several studies had confirmed the anti-inflammatory activities of indole-3-carbinol in a variety of animal models such as aspirin-induced gastric ulcer^17^, liver fibrosis^18^, lipopolysaccharide-induced acute lung injury^19^ and high fat diet-induced obesity^20^. In fact, indole-3-carbinol had been found to reduce the expression of pro-inflammatory cytokines such as IL-1β, IL-6, TNF-α and NF-kB^18^,^19^,^21^. Also, the expression of cyclooxygenase-2, lipoxygenase and inducible nitric oxide synthase enzymes was significantly reduced by indole-3-carbinol administration^22^,^23^. Moreover, indole-3-carbinol was shown to possess chemopreventive^24^ as well as anti-tumor activities^25^,^26^ via interference with a variety of signal transduction pathways involved in cell survival^23^.

Consequently, the present study was conducted to find answers to four raised questions: (1) Does indole-3-carbinol provide a significant nephroprotection against cisplatin-induced acute nephrotoxicity in rats? (2) If so, what are the possible mechanisms underlying this nephroprotective effect? (3) Is calcitonin gene-related peptide involved in the pathogenesis of cisplatin-induced renal injury and is it partially involved in indole-3-carbinol nephroprotection? And finally, (4) Does indole-3-carbinol exert any modulatory effect on cisplatin anti-cancer activity?

## Material and Methods

### Material

Indole-3-carbinol was purchased from Sigma Chemical Co. (St. Louis, MO, USA). The dose used was selected according to previous studies^16^,^17^. Besides, pilot experimental trials of the present study were carried out for the same purpose. Cisplatin was obtained from Merk Ltd. (Egypt). Cisplatin was given as a single dose of 7 mg/kg intraperitoneally^27^,^28^,^29^. Trichloroacetic acid, sulphorhodamine B dye and trizma base were obtained from Sigma Chemical Co. (St. Louis, MO, USA). All chemicals and solvents were of highest grade commercially available.

### Cell lines

Hela and PC3 human cancer cell lines were obtained frozen in liquid nitrogen from American Type Culture Collection (ATCC). The cell lines were maintained in Ain Shams University, Cairo, Egypt, by serial sub-culturing. Cells were grown as “monolayer culture” in RPMI-1640 medium supplemented with 10% (v/v) fetal bovine serum and 100 U/ml penicillin and 100 μg/ml streptomycin antibiotic. The cell lines were incubated at 37 °C in 5% CO_2_-95% air.

### Animals

Male albino rats (150–200 g) were obtained from Nile Co. for Pharmaceutical and Chemical industries (Egypt). The rats were housed in air-conditioned atmosphere, at a temperature of 25 °C with alternatively 12 h light and dark cycles. The animals were acclimated for 2 weeks before experimentation. They were kept on a standard diet and water *ad libitum*. Standard diet pellets (El-Nasr Chemical Company, Abu-Zaabal, Egypt) contained not less than 20% protein, 3.5% fat, 6.5% ash and a vitamin mixture.

### Ethics Statement

Animal care and all experimental protocol were approved and conducted in accordance with the guidelines approved by the Research Ethics Committee of Ain Shams University (REC-ASU), Egypt (Serial number of the protocol: Research after Ph.D. 3).

### *In vivo* part to assess the nephroprotective effects of indole-3-carbinol against cisplatin-induced acute nephrotoxicity in rats

#### Experimental design

Forty rats were divided randomly into four groups (n = 10) and treated for 2 weeks. Group 1 was given the vehicle daily and considered as control. Group 2 was given the vehicle once daily for 2 weeks starting 7 days before giving cisplatin (7 mg/kg i.p.) as a single dose to induce nephrotoxicity. Group 3 was given indole-3-carbinol at a dose of 20 mg/kg once daily for 2 weeks starting 7 days before giving a single dose of cisplatin (7 mg/kg i.p). Group 4 was given 20 mg/kg indole-3-carbinol once daily for 2 weeks. At the end of the experiment, blood samples were collected to measure levels of blood urea nitrogen and serum creatinine. Kidney tissues were dissected and homogenized to assess oxidative stress markers (reduced glutathione, lipid peroxides, superoxide dismutase as well as NADPH-oxidase-1), inflammatory markers (IL-1β and TNF-α), caspase-3 levels as well as the expression of growth factors (epidermal growth factor, insulin-like growth factor-1 and insulin-like growth factor-1 receptor). Also, the effect of different treatments on calcitonin gene-related peptide expression in tissues was determined. Moreover, histopathological examination was carried out on kidney specimens taken from the different treatment groups.

#### Assessment of nephrotoxicity markers

Levels of creatinine and urea in the serum obtained from the different treatment groups were determined using available commercial kits (Biodiagnotic Co., Egypt). Kidney index was calculated according to the formula: (kidney weight/total body weight) × 100.

#### Determination of platinum concentration in renal tissues

Tissues taken from the different treatment groups were dried at 85 °C in the oven overnight. Samples were digested with nitric acid, perchloric acid and hydrogen peroxide. Then, platinum concentration was determined using prodigy high dispersion inductively coupled plasma optical emission spectrometric method^29^,^30^.

#### Assessment of protein content

Protein contents in kidney homogenates were determined according to the method previously described by **Bradford**^31^ using bovine serum albumin as a standard.

#### Assessment of oxidative stress markers

Reduced glutathione tissue levels were meaured using reduced glutathione assay kit (Biodiagnostic, Egypt)^32^. Malondialdehyde assay kit (Biodiagnostic, Egypt) was used to assess lipid peroxidation. It was estimated by measuring the level of thiobarbituric acid reactive substances (TBARS) and the results were expressed as malondialdehyde equivalents^33^. The activity of superoxide dismutase in tissue homogenates was assessed using superoxide dismutase assay kit (Trevigen, Inc., USA). ELISA kit for NADPH oxdiase-1 (USCN Life Science Inc, China) was used to determine NADPH oxdiase-1 expression level in the different treatment groups. The manufacturer’s instructions were exactly followed.

#### Assessment of inflammatory markers

The effects of cisplatin and indole-3-carbinol on the renal expression of some pro-inflammatory mediators were examined. IL-1β and TNF-α levels in kidney tissue homogenate were assessed using Rat IL-1β and Rat TNF-α ELISA Kits (RayBiotech, Inc., USA), respectively. The steps were carried out according to the manufacturer’s instructions.

#### Determination of caspase-3 level

ELISA kit was used to assess caspase-3 level in kidney homogenates of the different treatment groups (USCN Life Science Inc, China). The procedure was carried out according to the manufacturer’s instructions.

#### Assessment of growth factors and calcitonin gene-related peptide expression

Effects of the different treatment groups on tissue expression of growth factors (epidermal growth factor, insulin-like growth factor-1 and insulin-like growth factor-1 receptor) and calcitonin gene-related peptide were determined using ELISA kits (USCN Life Science Inc, China). The steps were carried out according to the manufacturer’s instructions.

#### Histopathological examination

For light microscopy, autopsy samples were taken from the kidney of rats from the different groups and fixed in 10% formalin for 24 h. Washing was done in tap water then serial dilutions of alcohol (methyl, ethyl and absolute ethyl) were used for dehydration. Specimens were cleared in xylene and embedded in paraffin at 56 °C in hot air oven for 24 h. Paraffin bees wax tissue blocks were prepared for sectioning at 4 microns thickness by sledge microtome. The obtained tissue sections were collected on glass slides, deparaffinized, stained by hematoxylin & eosin stain for routine examination then examination was done using the light microscope^34^.

#### Immunohistochemical detection of NF-kB

Paraffin embedded tissue sections of 3 μm thickness were rehydrated first in xylene and then in graded ethanol solutions. The slides were blocked with 5% bovine serum albumin in tris buffered saline for 2 h. Then, the sections were immunostained with the primary rabbit polyclonal antibody to rat NF-kB (Santacruz Biotech, Cat No. sc-372) at a concentration of 1 μg/ml and incubated overnight at 4 °C. After washing the slides with tris buffered saline, the sections were incubated with the secondary antibody. Sections were then washed and incubated for 5–10 min in a solution of 0.02% diaminobenzidine containing 0.01% hydrogen peroxide. Counter staining was performed using hematoxylin and the slides were visualized under the light microscope.

### *In vitro* cytotoxicity assay

Cytotoxic activities of cisplatin and indole-3-carbinol were determined by sulphorhodamine B cytotoxicity assay^35^. Cancer cells were seeded in 96 well flat bottom plates in RPMI-1640 supplemented medium. Following 24 h of incubation, media was replaced with new media supplemented with appropriate drugs concentrations. Cisplatin was used at different concentrations ranging from 0.8 to 15 μg/ml and incubated for 24 h. Indole-3-carbinol was used at concentrations of 100, 200 and 400 μM. Following treatment, cells were fixed with 10% trichloroacetic acid for 1 h at 4 °C. Wells were washed with water and then stained with 50 μl 0.4% sulphorhodamine B in 1% acetic acid for 30 min at 25 °C. The dye was solubilized with 100 μl 10 mM trizma® base (pH 10.5). Optical density of each well was measured spectrophotometrically at 564 nm. Concentration-response curves were sketched and inhibitory concentration 50 for each curve was determined (Graph Pad, Prism software, version 5).

### *In vivo* assessment of anti-tumor activity

#### Tumor cells preparation and transplantation

The transplantable murine tumor cell line, namely Ehrlich ascites carcinoma cells, was obtained from National Cancer Institute, Cairo, Egypt. Ehrlich ascites carcinoma cells were maintained *in vivo* in female Swiss albino mice by intraperitoneal transplantation of 2 × 10^6^ cells/mouse every 10 days. Ascitic fluid was drawn from tumor-bearing mice at the log phase of the tumor cells (7–8th day of tumor bearing). The freshly drawn fluid was diluted with ice-cold sterile isotonic saline and each animal received 200 μl of tumor cell suspension containing 2 × 10^6^ tumor cells subcutaneously.

#### Experimental design of Ehrlich ascites carcinoma solid tumor model

Female Swiss albino mice weighing 20–25 g were divided into four groups (ten animals per group). When mice developed palpable mass (tumor volume range: 80–120 mm^3^), treatment was started (day 0). Group 1 was given the vehicle daily and considered as untreated control. Group 2 was given the vehicle once daily for 10 days and on day 7, cisplatin was injected at a single dose of 5 mg/kg i.p. Group 3 was given indole-3-carbinol at a dose of 20 mg/kg orally once daily for 10 days and on day 7, cisplatin was injected at a single dose of 5 mg/kg i.p. Group 4 was given 20 mg/kg indole-3-carbinol orally once daily for 10 days. The dose of cisplatin was selected according to previous studies^36^,^37^. Longest and shortest diameters of the tumor were measured using a digital Vernier caliper. Tumor volume of each animal was calculated using the following formula:


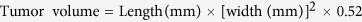


On day 11, animals were sacrificed and tumor specimens were excised and fixed in 10% formalin for histopathological examination using light microscopy. Also, Kidney specimens were taken for the assessment of histopathological alterations induced in the different treatment groups according to the method previously described.

### Statistical analysis

Data are presented as mean ± SD. Unpaired *t*-test was used to compare two different treatment groups. Multiple comparisons were performed using one-way ANOVA followed by Tukey Kramer as a post hoc test, as appropriate. The 0.05 level of probability was used as the criterion for significance. All statistical analyses were performed using Instat version 3 software package. Graphs were generated using GraphPad Prism (ISI®, USA) version 5 software.

## Results

### Nephrotoxicity markers

Table 1 shows the results of mortality percentage, total body weight, kidney index as well as renal function tests. In the control and indole-3-carbinol-only treated groups, no animal death was observed. Cisplatin-injected group showed the highest number of animal death where it reached 30%. Nevertheless, no animal death was recorded in indole-3-carbinol pre-treated rats. Regarding the change in body weight, cisplatin injection induced 17.5% decrease in the body weight as compared to the original body weight. Besides, kidney index was increased by 32.4% in cisplatin-treated group as compared to the control group. Moreover, serum creatinine and blood urea nitrogen were significantly increased in cisplatin-treated group by 744.7 and 387.6%, respectively when compared to the control values. However, pre-treatment with 20 mg/kg indole-3-carbinol significantly reduced serum creatinine and blood urea nitrogen levels by 55.8 and 63.2%, respectively when compared to the cisplatin group. Furthermore, there was no significant change in nephrotoxicity indices in indole-3-carbinol only-treated groups when compared to the control group.

### Determination of platinum concentration in kidney tissue

Pre-treatment with 20 mg/kg indole-3-carbinol induced no significant change in renal platinum concentration in comparison with the cisplatin group. Besides, platinum levels were undetectable in the control and indole-3-carbinol only-treated rats (data not shown).

### Assessment of oxidative stress markers

The tissue levels of reduced glutathione, malondialdehyde, superoxide dismutase and NADPH oxidase-1 in the different treatment groups are shown in Table 2. Cisplatin induced a marked depletion in the antioxidant capacity where reduced glutathione and superoxide dismutase levels were significantly reduced by 84.2 and 76.5% as compared to the control values. Also, malondialdehyde and NADPH oxidase-1 level were significantly increased in cisplatin-injected group by 612.6 and 420.9%, respectively when compared to the control group. Nevertheless, the group pre-treated with 20 mg/kg indole-3-carbinol showed a 4.5- and 4.7-fold increase in reduced glutathione and superoxide dismutase tissue levels, respectively as compared to the cisplatin group. Regarding malondialdehyde and NADPH oxidase-1, their tissue levels were reduced by 78.9 and 75.7%, respectively in the indole-3-carbinol pre-treated group as compared to the cisplatin group.

### Assessment of pro- inflammatory markers

The effects of cisplatin and indole-3-carbinol on IL-1β and TNF-α levels in kidney tissue homogenates of the different treatment groups were determined (Table 2). Cisplatin induced a 10.4- and 7.8-fold increase in IL-1β and TNF-α tissue levels, respectively as compared to the control values. On the other hand, pre-treatment with indole-3-carbinol (20 mg/kg) counteracted the increase in IL-1β and TNF-α tissue levels where they were reduced by 83.7 and 71.7%, respectively when compared to the cisplatin-injected group.

**Figure 1:**
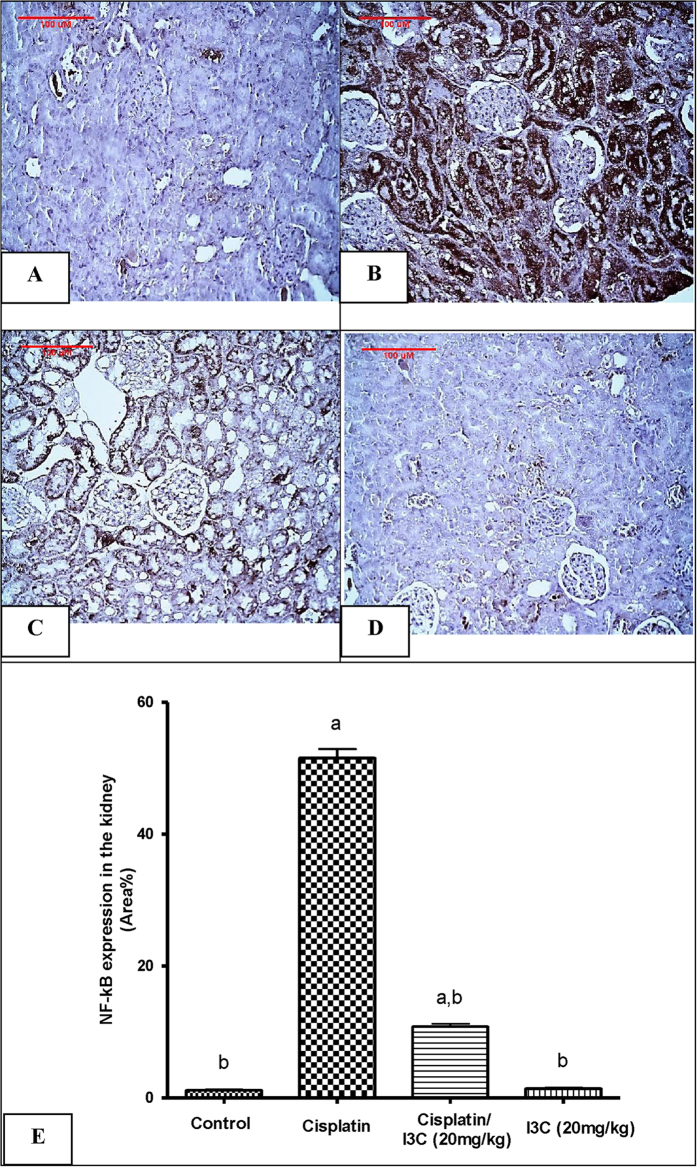
Expression of Nuclear factor-kappa B by immunohistochemical staining. (**A**) Control group shows a minimal expression of NF-kB. (**B**) Cisplatin-injected group shows an extensive NF-kB expression (brown staining). (**C**) Indole-3-carbinol pre-treated group shows a limited NF-kB expression. (**D**) Indole-3-carbinol-only treated group showing a minimal NF-kB expression. (**E**) Area percentage of immuno-positive reaction. Values are given as mean ± SD for each group. a or b: Statistically significant from the control or the cisplatin group, respectively, P < 0.05 using ANOVA followed by Tukey-Kramer as post-hoc test.

Moreover, the immunohistochemical analysis of NF-kB tissue expression revealed that its expression was markedly induced by cisplatin administration reaching 44.1-fold, as compared to the control values that was evident by the extensive brown staining (Fig. 1B). In contrast, this elevation was significantly reduced by 79% in the group pre-treated with indole-3-carbinol as compared to the cisplatin group (Fig. 1C). However NF-kB tissue expression was minimal in both the control and indole-3-carbinol-only groups (Fig. 1A,D).

### Assessment of the level of caspase-3 colorimetrically

**Figure 2:**
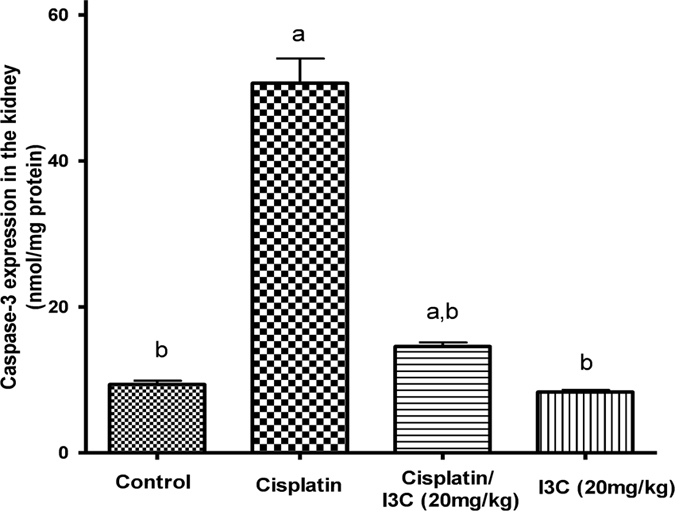
Effects of cisplatin and indole-3-carbinol on the expression of caspase-3 in kidney tissues of rats. a or b: Statistically significant when compared to the control or the cisplatin group, respectively, P < 0.05 using ANOVA followed by Tukey-Kramer as post-hoc test.

It was found that caspase-3 tissue level was significantly increased by cisplatin injection by 5.4 folds, respectively as compared to the control values (Fig. 2). Increased level of caspase-3 was significantly attenuated by indole-3-carbinol pre-treatment reaching 71.23%, as compared to the cisplatin group. No significant change in caspase-3 expression was evident in indole-3-carbinol-only treated group as compared to the control group.

### Growth factors expression

**Figure 3:**
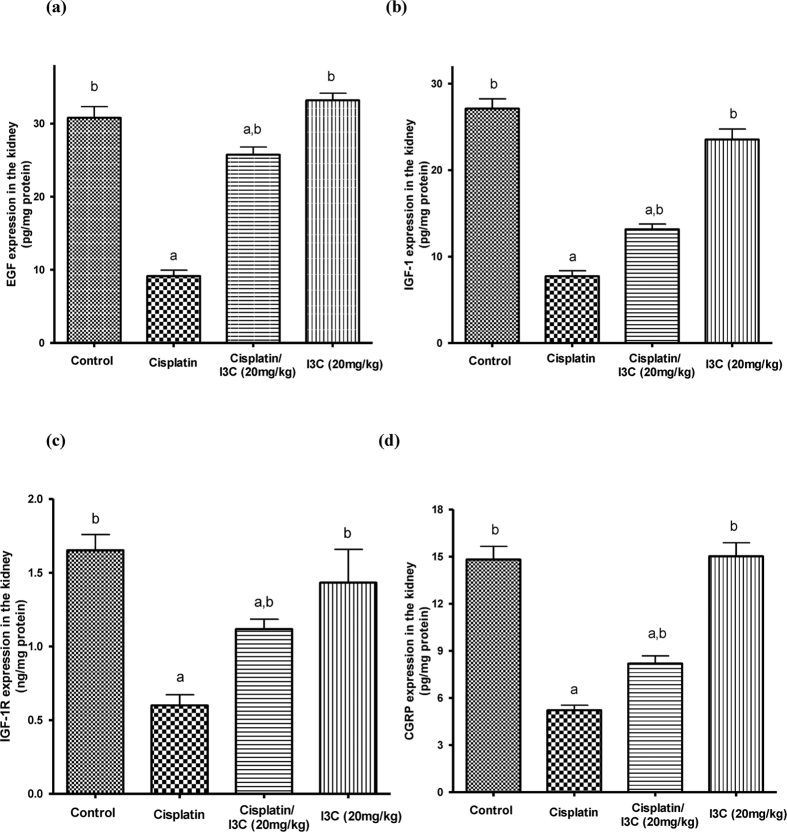
Effects of cisplatin and indole-3-carbinol on the expression of growth factors and calcitonin gene-related peptide in kidney tissues of rats. a or b: Statistically significant when compared to the control or the cisplatin group, respectively, P < 0.05 using ANOVA followed by Tukey-Kramer as post-hoc test. EGF: epidermal growth factor; IGF-1: insulin-like growth factor-1; IGF-1R: insulin-like growth factor-1 receptor; CGRP: calcitonin gene-related peptide.

Notably, cisplatin injection significantly reduced epidermal growth factor and insulin-like growth factor-1 expression by 70.3 and 71.4% as compared to the control values. However, pre-treatment with 20 mg/kg indole-3-carbinol induced a 2.7- and 1.7-fold increase in their tissue levels, respectively as compared to the cisplatin group (Fig. 3A,B). In addition, indole-3-carbinol significantly attenuated the decrease in insulin-like growth factor-1 receptor expression induced by cisplatin administration where its expression was increased in the pre-treated group by 86.3% as compared to the cisplatin group (Fig. 3C). Besides, there was no significant change in the expression of the assessed growth factors in indole-3-carbinol-only treated group as compared to the control values.

### Calcitonin gene-related peptide expression

Assessment of calcitonin gene-related peptide expression levels in renal tissues revealed that cisplatin injection induced a significant decrease in its expression by 64.8% as compared to the control. This effect was partially reversed by 57.1% in indole-3-carbinol pre-treated group as compared to the cisplatin group (Fig. 3D).

### Kidney histopathological assessment of the model of cisplatin-induced acute nephrotoxicity in rats

**Figure 4:**
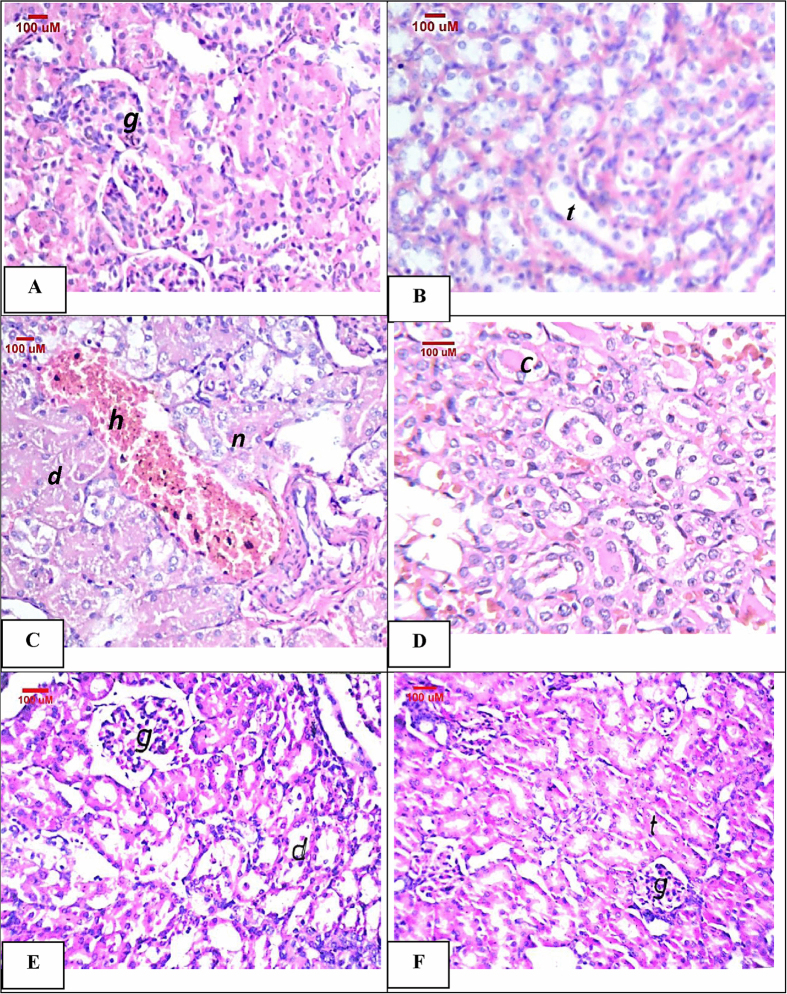
Representative photomicrographs of kidney sections stained by H&E: (**A**,**B**) Control group showing normal glomeruli (g) and normal histological structure of the tubules (t). (**C**,**D**) Cisplatin-injected group showing necrosis (n), degeneration (d) and cystic dilatation of the tubules with focal hemorrhages (h) in between. Besides, homogenous eosinophilic casts in lumen of medullary tubules (c) were shown. (**E**) Indole-3-carbinol pre-treated rats showing degeneration (d) in the lining epithelium of the tubules. (**F**) Indole-3-carbinol only-treated rats showing normal histological structures.

Figure 4 shows the histopathological alterations in kidney specimens taken from the different treatment groups. No histopathological alterations were observed in kidney sections taken from the control rats (Fig. 4A,B). However, cisplatin injection induced a very severe degree of degeneration and necrosis in the lining epithelium of the renal tubules together with homogenous eosinphilic casts in the lumen of the tubules at the medullary portion. Besides, cystic dilatation and focal hemorrhages were observed (Fig. 4C,D). On the contrary, indole-3-carbinol pre-treatment significantly attenuated cisplatin-induced histopathological changes where some of the tubules showed moderate degenerative change in the lining epithelium, while necrosis was not observed (Fig. 4E). Moreover, normal histological structures of the glomeruli and tubules were observed in the kidney sections taken from the indole-3-carbinol-only treated group (Fig. 4F).

### Modulatory effect of I3C pre-treatment on cisplatin cytotoxic activity in human cancer cell lines

**Figure 5:**
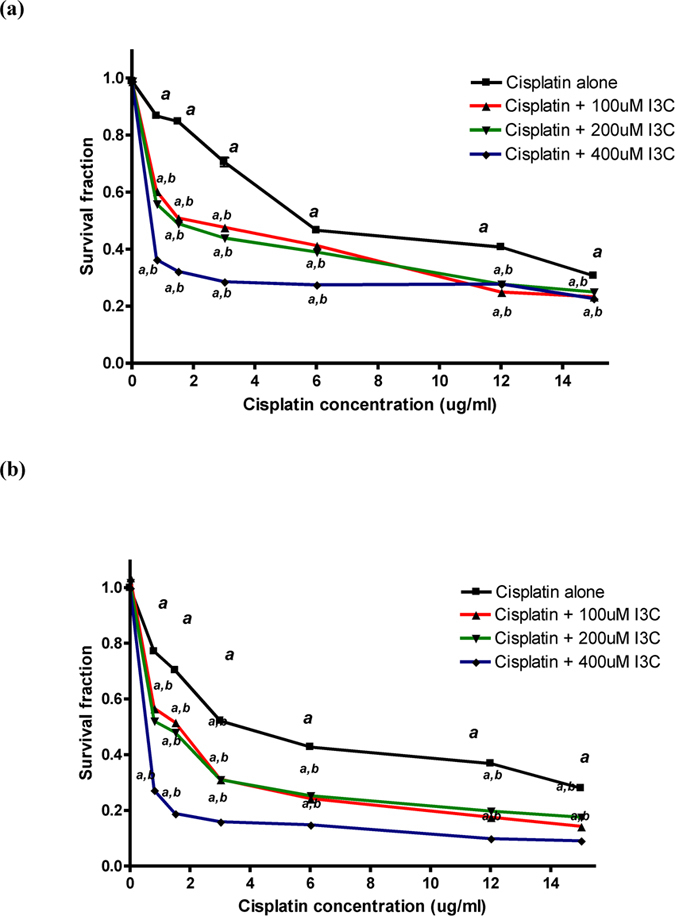
Cytotoxicity of various concentrations of cisplatin alone or in combination with indole-3-carbinol in hela cancer cell line (**a**) and PC3 cancer cell line (**b**). ^a,b^p < 0.05: Statistically significant when compared to the control or the corresponding group treated with cisplatin alone, respectively, P < 0.05 using ANOVA followed by Tukey-Kramer as post-hoc test.

Figure 5 shows cell viability expressed in terms of survival fraction as compared to the untreated control cells using sulphorhodamine B cytotoxicity assay. Twenty four hour treatment with cisplatin induced a significant cytotoxicity in both hela and PC3 human cancer cells, in a dose-dependent manner. Inhibitory concentration 50 of cisplatin was found to be 5.46 and 3.65 μg/ml for hela and PC3 cancer cells, respectively. However, pre-treatment with indole-3-carbinol at concentrations of 100, 200 and 400 μM for 24 h significantly enhanced cisplatin cytotoxicity in hela and PC3 cancer cells, in a dose dependent manner where using indole-3-carbinol at a concentration of 400 μM reduced cisplatin inhibitory concentration 50 significantly to 0.59 and 0.49 μg/ml for hela and PC3 cancer cells, respectively.

### *In vivo* assessment of anti-tumor activity of indole-3-carbinol and cisplatin in Ehrlich ascites carcinoma solid tumor model in mice

**Figure 6:**
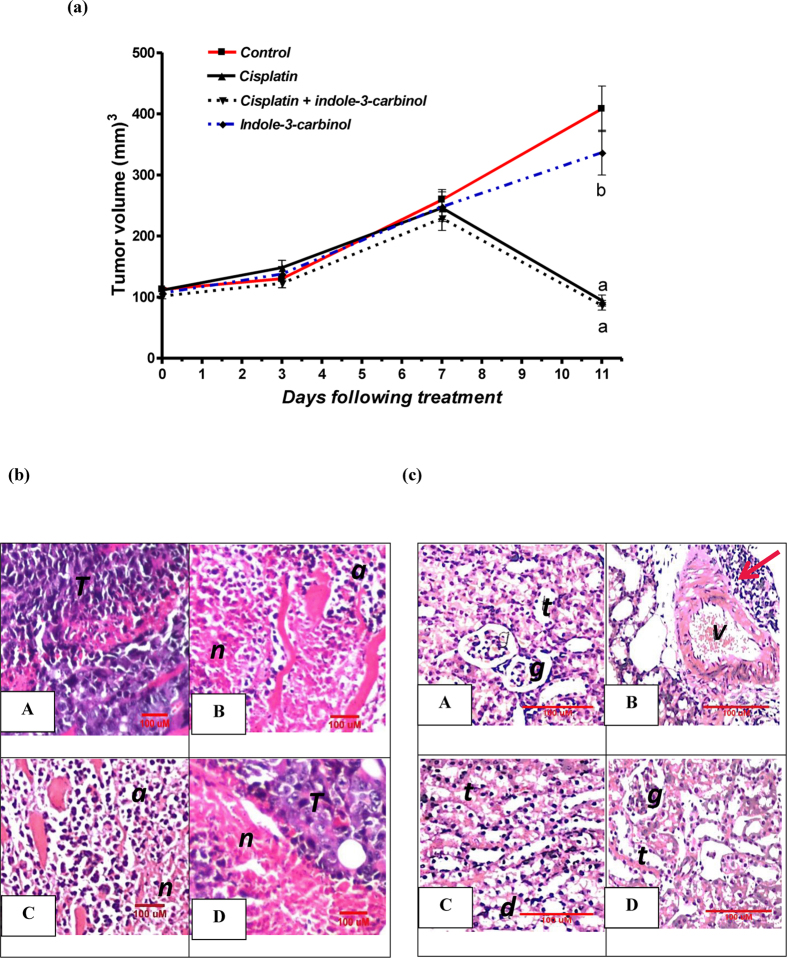
(**A**) Effects of cisplatin and/or indole-3-carbinol treatment on tumor growth in Ehrlich ascites carcinoma solid tumor model in mice. a or b: Statistically significant when compared to the control or the cisplatin group, respectively, P < 0.05 using ANOVA followed by Tukey-Kramer as post-hoc test. (**B**) Representative photomicrographs of Ehrlich ascites carcinoma solid tumor sections taken from the different treatment group and stained by H&E. T: intact structure of tumor cells. n: necrosis of tumor cells. a: apoptosis of tumor cells. (**C**) Representative photomicrographs of H&E-stained kidney sections taken from the different groups in Ehrlich ascites carcinoma solid tumor model in mice. A: Untreated mouse bearing Ehrlich ascites carcinoma solid tumor showing normal glomeruli (g) and tubules (t) at the renal cortex. B: Ehrlich ascites carcinoma solid tumor-bearing mouse injected with cisplatin (5 mg/kg) showing degeneration together with focal aggregation of lymphoid cells (red arrow) surrounding the congested blood vessel (v). (**C**) Ehrlich ascites carcinoma solid tumor-bearing mouse treated with cisplatin and indole-3-carbinol showing degeneration (d) in the epithelial lining of the tubules (t). (**D**) Ehrlich ascites carcinoma solid tumor-bearing mouse treated with indole-3-carbinol alone showing normal glomeruli (g) and tubules (t).

On day 11, tumor volume in untreated Ehrlich ascites carcinoma cells bearing-mice was significantly increased by 3.6 folds as compared to the tumor volume observed on day 0. In contrast, groups treated with cisplatin alone or in combination with indole-3-carbinol showed a significant reduction in tumor volume by 15.2 and 14.7%, respectively, as compared to that observed on day 0. However, treatment with indole-3-carbinol alone did not show any significant change in tumor volume as compared to the untreated control group (Fig. 6A). Moreover, these findings were supported by the histological assessment of tumor specimens taken from the different treatment groups. In the untreated control group, tumor cells were intact and grouped. On the other hand, groups given cisplatin alone or in combination with indole-3-carbinol showed severe apoptosis as well as necrosis. While, in the group given indole-3-carbinol only, areas of mild necrosis were detected in between intact tumor cells (Fig. 6B).

### Histopathological assessment of cisplatin nephrotoxicity in Ehrlich ascites carcinoma solid tumor model in mice

Kidney specimens were taken from the different treatment groups in Ehrlich ascites carcinoma solid tumor model to evaluate the effect of cisplatin and indole-3-carbinol on renal histological structure. Both the untreated control and the indole-3-carbinol-only treated groups showed normal histological structures of glomeruli and tubules at the renal cortex. Notably, sections taken from the cisplatin-injected rats showed congested blood vessels and focal degeneration in the corticomedullary tubules. Besides, focal aggregation of inflammatory cells surrounding the congested blood vessels was observed. Nevertheless, in the group pre-treated with indole-3-carbinol, mild degeneration in the epithelial cells lining the tubules was observed (Fig. 6C).

## Discussion

The current study was the first one to explore the potential protective effect of indole-3-carbinol against cisplatin-induced acute nephrotoxicity in rats. Besides, the possible mechanisms underlying this nephroprotective effect were investigated including its effects on oxidative and inflammatory status as well as apoptosis. Interestingly, the possible roles of insulin-like growth factor-1, insulin-like growth factor receptor-1 and calcitonin gene-related peptides in cisplatin-induced renal damage were explored. In addition, the possible modulatory effect of indole-3-carbinol pre-treatment on the anticancer activity of cisplatin was investigated *in vitro* in hela and PC3 human cancer cell lines as well as in Ehrlich ascites carcinoma solid tumor model in mice.

To induce acute nephrotoxicity in rats, cisplatin was injected intraperitoneally as a single dose of 7 mg/kg. As compared to the control group, cisplatin-injected rats showed a significant decrease in total body weight and a significant increase in kidney index as well. Besides, nephrotoxicity indices including mortality rate, blood urea nitrogen and serum creatinine were significantly increased indicating acute nephrotoxicity. These findings confirmed those of previous studies^27^,^28^,^29^. Notably, pre-treatment with 20 mg/kg indole-3-carbinol had significantly improved nephrotoxicity indices. Indeed, histopathological examination showed only moderate degeneration in the tubular lining epithelium in the indole-3-carbinol pre-treated group.

This was followed by investigating the possible mechanisms underlying the nephroprotective effects of indole-3-carbinol. There is strong evidence that depletion of intracellular anti-oxidants as well as generation of reactive oxygen and nitrogen species play a crucial role in the pathogenesis of cisplatin-induced nephrotoxicity^12^,^38^. This study showed that cisplatin induced a marked oxidative stress in kidney tissues where reduced glutathione levels and superoxide dismutase were significantly reduced, while lipid peroxidation was significantly increased as compared to the control group. These findings were in accordance with those demonstrated by **Arjumand and Sultana**^28^ **and El-Naga**^29^. Moreover, cisplatin-injected group showed a significant increase in NADPH oxidase-1. NADPH oxidases is a group of enzymes that have been found to be involved in many pathological conditions as they have reactive oxygen species generating capacities^39^. Moreover, NADPH oxidase-1 enzyme has been reported to be involved in cisplatin-induced ototoxicity^40^ as well as nephrotoxicity^29^,^41^. In this study, indole-3-carbinol pre-treatment markedly ameliorated cisplatin-induced oxidative stress where tissue levels of glutathione levels, superoxide and NADPH oxidase-1 nearly returned to basal control levels and lipid peroxidation was significantly reduced as well. Indeed, anti-oxidants showed to have a potential protective effect against cisplatin-induced renal injury^29^,^41^,^42^.

In addition to the role of oxidative stress, inflammation is critically involved in the pathogenesis of cisplatin-induced renal injury^3^. Besides, using anti-inflammatory agents have been found to significantly attenuate cisplatin-induced nephrotoxicity^29^,^43^,^44^. In this context, using a TNF-α inhibitors increased the survival of mice given cisplatin by mitigating its toxicity^45^. In the present study, cisplatin injection significantly increased tissue levels of pro-inflammatory markers; IL-1β, TNF-α and NF-kB. However, pre-treatment with indole-3-carbinol exerted a marked anti-inflammatory activity, where the levels of pro-inflammatory markers were significantly reduced when compared to the cisplatin group. Several studies had shown that indole-3-carbinol has a marked anti-inflammatory properties *in vitro*^18^,^19^ as well as *in vivo*^18^,^20^,^21^. Accumulation of platinum was found to play a role in cisplatin-induced nephrotoxicity^46^,^47^. However, reducing platinum uptake was not found to be contributing to the nephroprotective effects conferred by indole-3-carbinol.

Previous studied showed that both intrinsic and extrinsic apoptotic pathways are involved in cisplatin-induced nephrotoxicity^48^,^49^. Levels of proteolytic caspases and other apoptotic proteins such as p53 were found to be increased by cisplatin both *in vivo*^41^ and *in vitro*^50^. Also, DNA fragmentation, the hallmark of apoptosis, was evident in cisplatin-treated animals^51^. Our findings showed that the expression of caspase-3 enzyme was significantly elevated in the cisplatin-treated group. Nevertheless, these effects were reversed in the indole-3-carbinol pre-treated group. Moreover, depletion of growth factors and their receptors have been recently shown to play a role in cisplatin-induced toxicities. In a rat model, cisplatin resulted in a down-regulation of epidermal growth factor/epidermal growth factor receptor pathway^52^. Also, **Yasuda** *et al*.^53^, demonstrated that the exogenous administration of recombinant human insulin like growth factor attenuated renal damage induced by cisplatin in rats. This study was the first one to provide evidence that insulin like growth factor-1 as well as insulin like growth factor-1 receptor are down-regulated by cisplatin-injection, an effect which was significantly attenuated by indole-3-carbinol pre-treatment.

Indeed, cisplatin was shown to alter renal vasculature hemodynamics where intraperitoneal injection of sildenafil, phosphodiesterase-5 inhibitor, showed a marked protection against cisplatin-induced nephrotoxicity^54^. Also, the modulatory role of nitric oxide in cisplatin-induced renal damage is well established^27^. Calcitonin gene-related peptide is a potent vasodilator neuropeptide which is known to be involved in pain transmission, migraine and cardiovascular homeostasis. Receptors of calcitonin gene-related peptide are not restricted to the CNS, but expressed throughout the body to regulate many physiological functions^55^. Calcitonin gene-related peptide affects renal hemodynamics by increasing renal cyclic AMP levels in a dose-dependent manner both *in vitro*^56^ and *in vivo*^57^. Interestingly, Li *et al*.^58^ found that calcitonin gene-related peptide exerts a nephroprotective effect in a model of deoxycorticosterone-salt hypertension in mice. In this context, carvedilol had been shown to reduce blood pressure in spontaneous hypertensive rats partially by increasing renal expression of calcitonin gene-related peptide and hence increasing renal blood flow^59^. However, the role of calcitonin gene-related peptide in cisplatin-induced nephrotoxicity was not investigated before. The present study was the first one to demonstrate that calcitonin gene-related peptide expression was significantly decreased in cisplatin-injected rats; however, its level was nearly normalized in the indole-3-carbinol pre-treated group.

Besides, the possible modulatory effect of indole-3-carbinol on cisplatin cytotoxic activity was very important to be investigated *in vitro* where two human cancer cell lines; hela and PC3, were used. Notably, it was found that pre-treatment with indole-3-carbinol for 24 h significantly enhanced cisplatin cytotoxicity in the two cancer cell lines in a dose-dependent manner. In agreement with this, a derivative of indole-3-carbinol showed a synergistic effect with cisplatin in human breast cancer cells *in vitro*^60^. Moreover, it was found that in Ehrlich ascites carcinoma solid tumor model, the mice that received treatment with cisplatin and indole-3-carbinol showed a significant reduction in tumor volume as compared to the untreated tumor-bearing mice, a result which was further confirmed by histopathological assessment.

In conclusion, this was the first study to demonstrate that indole-3-carbinol pre-treatment provides a marked nephroprotection against cisplatin-induced acute nephrotoxicity. This nephroprotective effect could be partially attributed to its anti-oxidant activities manifested by significantly increasing reduced glutathione and superoxide dismutase as well as decreasing lipid peroxidation levels. Also, indole-3-carbinol was shown to be a powerful NADPH oxidase-1 inhibitor where it reversed cisplatin-induced increase in NADPH oxidase-1 expression. This finding may pave the way to investigate the effects of indole-3-carbinol in other pathological conditions where NADPH oxidase-1 is invoved. In addition, indole-3-carbinol had been shown to markedly attenuate cisplatin-induced inflammatory response via reducing tissue levels of pro-inflammatory markers; IL-1β, TNF-α and NF-kB. Moreover, indole-3-carbinol counteracted cisplatin-induced renal cell death via reducing caspase-3 levels and increasing the expression of growth factors; epidermal growth factor, insulin-like growth factor-1 and insulin-like growth factor-1 receptor as well. Additionally, this study shed the light on the possible role of calcitonin gene-related peptide down-regulation in cisplatin nephrotoxicity which was reversed by indole-3-carbinol pre-treatment. Notably, using indole-3-carbinol preserved cisplatin anti-cancer activity both *in vitro* in two human cancer cell lines and *in vivo* in Ehrlich ascites carcinoma solid tumor model. Collectively, our findings open a new avenue for using indole-3-carbinol to improve the therapeutic index of cisplatin.

## Additional Information

**How to cite this article**: El-Naga, R. N. and Mahran, Y. F. Indole-3-carbinol protects against cisplatin-induced acute nephrotoxicity: role of calcitonin gene-related peptide and insulin-like growth factor-1. *Sci. Rep.* **6**, 29857; doi: 10.1038/srep29857 (2016).

## Figures and Tables

**Table 1:** Effects of cisplatin and indole-3-carbinol on nephrotoxicity markers in rats.

Treated groups	No of dead rats	Body weight (g)		Relative kidney weight	Blood urea nitrogen (mg/dl)	Serum creatinine (mg/dl)
		Before treatment	After treatment			
Control	0/10	183.80 ± 8.5	228.30 ± 9.4^*^	0.71 ± 0.05^b^	35.75 ± 3.7^b^	0.38 ± 0.12^b^
Cisplatin	3/10	185.00 ± 18.7	152.60 ± 10.4^*^	0.94 ± 0.20^a^	174.33 ± 10.82^a^	3.21 ± 0.85^a^
Cisplatin/indole-3-carbinol (20 mg/kg)	0/10	174.00 ± 13.5	172.00 ± 13.7	0.57 ± 0.10^b^	64.17 ± 14.50^a,b^	1.42 ± 0.16^a,b^
Indole-3-carbinol (20 mg/kg)	0/10	178.30 ± 12.6	195.00 ± 8.9	0.74 ± 0.10^b^	34.83 ± 2.40^b^	0.46 ± 0.05^b^

All nephrotoxicity markers were assessed at the end of the experiment (7 days following cisplatin single dose injection).

Data are mean ± SD (n = 6–10).

a or b: Statistically significant when compared to the control or cisplatin group, respectively, P < 0.05 using ANOVA followed by Tukey-Kramer as post-hoc test.

^*^Statistically significant when compared to the values obtained before treatment, P < 0.05 using unpaired t-test.

**Table 2:** Effects of cisplatin and indole-3-carbinol on the markers of oxidative stress and inflammation in kidney tissues of rats.

Treated groups	GSH(μmol/g tissue)	MDA (nmol/g tissue)	SOD (U/mg protein)	NOX-1 (ng/mg protein)	IL-1β (pg/mg protein)	TNF-α (pg/mg protein)
Control	2.91 ± 0.12	12.20 ± 0.96	13.02 ± 1.84	1.39 ± 0.35	13.03 ± 0.97	12.33 ± 1.26
Cisplatin	0.46 ± 0.11^a^	86.93 ± 7.57^a^	3.06 ± 0.69^a^	7.24 ± 0.88^a^	135.00 ± 10.34^a^	95.63 ± 9.22^a^
Cisplatin/indole-3-carbinol (20 mg/kg)	2.07 ± 0.31^a,b^	18.30 ± 2.3^b^	14.43 ± 1.24^b^	1.76 ± 0.33^b^	22.03 ± 2.75^a,b^	27.02 ± 6.30^a,b^
Indole-3-carbinol (20 mg/kg)	2.75 ± 0.26^b^	12.43 ± 0.81^b^	19.48 ± 1.99^a,b^	0.77 ± 0.67^b^	12.43 ± 0.81^b^	12.18 ± 2.78^b^

Data are mean ± SD (n = 6).

a or b: Statistically significant when compared to the control or cisplatin group, respectively, P < 0.05 using ANOVA followed by Tukey-Kramer as post-hoc test.

GSH: reduced glutathione; MDA: malondialdehyde; SOD: superoxide dismutase; NOX-1: NADPH oxidase-1; IL-1β: interleukin-1 beta; TNF-α: tumor necrosis factor-alpha.
